# Editorial: Mobile DNA element-driven evolution of bacterial pathogens

**DOI:** 10.3389/fmicb.2025.1583263

**Published:** 2025-03-19

**Authors:** Axel Cloeckaert, Michel S. Zygmunt, Filipa F. Vale, Eric Altermann

**Affiliations:** ^1^INRAE, Université de Tours, UMR, ISP, Nouzilly, France; ^2^Pathogen Genomics and Translational Microbiology Lab, BioISI – Instituto de Biosistemas e Ciências Integrativas, Faculdade de Ciências, Universidade de Lisboa, Lisbon, Portugal; ^3^Massey University, School of Veterinary Science, Palmerston North, New Zealand; ^4^Blue Barn Life Sciences, Ltd, Feilding, New Zealand

**Keywords:** insertion sequence, bacterial pathogen, genome evolution, chromosome, plasmid, antimicrobial resistance, virulence, adaptation

Mobile DNA elements, such as Insertion Sequence (IS) elements, transposons, integrative elements, and prophages are key players in bacterial adaptation and evolution (Ghaly and Gillings, 2018; Gomberg and Grossman, 2024; Harmer and Hall, 2024; Vos et al., 2024). They are currently classified in numerous families, the representatives of which may be genus- or species-specific. Through their mobility by transposition or integration they shape the bacterial genome and contribute to the adaptation of bacteria to survive changing environmental conditions or to adapt to animal or human hosts and evolve to a pathogenic status. They are also involved in the uptake of foreign DNA via horizontal gene transfer (HGT), ranging in size and function from single genes to pathogenicity islands, or islands providing new functional and metabolic characteristics, such as changes to the bacterial cell surface. The action of mobile DNA elements and associated HGT may therefore drive evolutionary pathogenic processes that include altered responses to inflammatory markers and the evasion of the host immune system.

Mobile DNA elements are also major contributors to the spread of antimicrobial resistance genes, some of which are known to mobilize intrinsic chromosomal genes to major carriers of antimicrobial resistance such as plasmids or genomic islands. In addition, IS elements may carry promoter sequences that can activate the expression of silent genes following their transposition, and through regulatory interference, contribute to enhanced resistance to environmental factors such as antimicrobials or other anti-bacterial factors within the host. IS-induced enhanced resistance may be the result of overexpression of efflux systems, decreased outer membrane permeability or modulated biofilm formation. High throughput sequencing has revealed numerous mobile DNA elements in the bacterial world, many of which remain to be functionally characterized.

The present Research Topic focused on mobile DNA elements and their contribution to pathogen evolution, including their acquisition of pathogenicity and/or antimicrobial resistance characteristics.

Twelve articles were published within this Research Topic in five different sections of Frontiers in Microbiology, namely “Antimicrobials, Resistance and Chemotherapy,” “Infectious Agents and Disease,” “Food Microbiology,” “Microbiotechnology,” and “Phage Biology.” This Research Topic highlights the interest in studying mobile DNA as a common theme across a wide range of impact areas.

The majority of articles included here focus on antimicrobial resistance, with six articles published in the section “Antimicrobials, Resistance and Chemotherapy,” one published in the section “Infectious Agents and Disease,” and one published in the section “Food Microbiology,” again demonstrating the interest in this important theme across the different sections of Frontiers in Microbiology. The majority of articles address specific pathogens (at the genus or species level) and two aim at assessing the resistome in (i) non-pathogenic bacterial species used in food products and (ii) pathogenic or non-pathogenic species found in the aquatic environment.

The following articles have been published in the Antimicrobials, Resistance and Chemotherapy section. Wang, Zhu, et al. investigated class 1 integrons and multiple mobile genetic elements in clinical isolates of the *Klebsiella pneumoniae* complex from a tertiary hospital in Eastern China. Using whole genome sequencing of 167 isolates, the authors identified a total of 169 antibiotic resistance gene cassettes encoding 19 types of resistance genes, including important carbapenem and class D beta-lactamase genes. Of particular interest, a duplicated region of 19 kb on one plasmid carrying an IS*26*-Int1 complex multidrug resistance integron was identified, which constitutes a new structure of a mobile genetic element involved in the spread of antibiotic resistance. In another study, Wang, Shen, et al. reported the mobilization of the carbapenem resistance gene *bla*_KPC − 14_ among heterogeneous plasmids in extensively drug-resistant hypervirulent *K. pneumoniae*. This resistance gene was located on an IncFII/IncR plasmid within a genetic structure, called the NTE_KPC_-Ib element, consisting of the *bla*_KPC − 14_ gene flanked by two IS elements, IS*Kpn27* and IS*Kpn6*. The authors assessed the horizontal transferability of the integrated NTE_KPC_-Ib plasmids and concluded that *bla*_KPC − 14_ is prone to integrate into other conjugative plasmids through this mobile DNA element. Also with regard to multidrug resistance in opportunistic bacteria, Mei et al. characterized a 522 kb mobilizable megaplasmid carrying a 93.5 kb multiple antibiotic resistance region, including *mer* operons conferring heavy metal mercury resistance, from a clinical *Pseudomonas aeruginosa* isolate. A bioinformatic analysis further revealed that many functional genes are flanked by IS elements that may have accumulated in the megaplasmid following multiple acquisition events. Focusing on enteric pathogens, Peng et al. reported the emergence of the fourth mobile sulfonamide resistance gene *sul4* in clinical *Salmonella enterica*. The authors showed that this resistance gene was carried by a complex chromosomally integrated hybrid plasmid. Regarding its mobilization, an IS*CR20*-like element was found to be associated with *sul4*. Of interest in the field of antibiotic resistance reservoirs, Kaszab et al. investigated the resistome of Lactobacilliales by analyzing whole genome sequences available in the NCBI RefSeq database. These bacteria are commonly used in food products and as probiotics in veterinary and human medicine. They are considered safe but may nevertheless carry antibiotic resistance genes (ARGs) that can be transferred to human or veterinary pathogens, raising veterinary and public health concerns. The authors screened the database for ARGs and assessed the possibility of their transmissibility by plasmid transfer or by linkage to integrative mobile genetic elements. The most prevalent transferable ARGs appeared to be *tet*M and *tet*W, which confer resistance to tetracycline. Although not as critical as the resistance genes found in pathogenic species cited in the other studies above, this study highlighted the One Health concept by demonstrating the potential for Lactobacilliales to serve as reservoirs for transferable ARGs. Regarding the aquatic environment resistome, Jiao et al. provided new insights into the microbiome, resistome, and mobilome of dental wastewater in the context of a heavy metal environment. Among hospital wastewater, dental wastewater contains heavy metals that may contribute to the development of antimicrobial resistance in this aquatic environment. The authors identified numerous ARGs, such as those that confer multidrug resistance and resistance to antibiotics that are frequently used in clinical practice. The main bacterial species identified as harboring these ARGs were *P. aeruginosa, Pseudomonas putida, Chryseobacterium indologenes*, and *Sphingomonas lateriae*. Along with the ARGs many mobile genetic elements were detected, IS elements and transposons, highlighting their potential role in the mobilization of ARGs as in the studies cited above.

In the Infectious Agents and Disease section, Glambek et al. reported on antimicrobial resistance patterns in *Streptococcus dysgalactiae* from a One Health perspective. This bacterial species is an important pathogen in both humans and a wide range of animal species and is therefore of interest from a One Health point of view. The authors investigated, using whole genome sequencing and antimicrobial susceptibility testing, the zoonotic potential of *S. dysgalactiae* and the exchange of antimicrobial resistance traits between different host populations carrying this pathogen in Norway. The authors provided evidence for niche specialization with respect to the distribution of resistance genes and mobile genetic elements, associated with a specific phylogenetic distribution, in isolates from infected humans (*n* = 274, bloodstream infections) and from infected animals (*n* = 133). For example, the erythromycin resistance gene *erm*(A) appeared dominant in human isolates, whereas *erm*(B) and *lsa*(C) were only identified in animal isolates. The *tet*(O) tetracycline resistance gene was located on distinct mobile elements between animal and human isolates. Common mobile elements were observed in only four isolates from different host species including one human, among the total of 407 isolates investigated. In conclusion, this study suggests that *S. dysgalactiae* has evolved into host-adapted populations and niche specialization, and direct exchange of strains or genetic elements from different ecological niches appears to be rare, at least in the geographical region of Norway investigated.

In the Food Microbiology section, Bartsch et al. characterized multidrug-resistant (MDR) *Salmonella enterica* serovar Agona isolates from a dietary supplement in Germany. This study involved serovar Agona isolates that appeared phylogenetically distinct from others available in databases and aimed thus to find a potential reservoir of this MDR strain and associated mobile genetic elements conferring MDR. Whole genome sequencing revealed the presence of 23 different ARGs conferring resistance to 12 different classes of antibiotics, together with genes conferring resistance to six different heavy metals. A large plasmid of 295 kb belonging to the IncHI2 plasmid family was shown to carry 16 ARGs, organized in two clusters. Each ARG was associated with putative composite transposons. A database search further revealed that similar plasmids are found in *Salmonella* isolates from a wide variety of livestock and in other bacterial genera from different geographical origins and isolation sources. In other words, the host range of this MDR plasmid appears to be broad and has already spread into different bacterial populations, highlighting the need for continuous surveillance of MDR foodborne pathogens such as *Salmonella* spp.

In addition to the articles on antimicrobial resistance cited above, two articles published in the Infectious Agents and Disease section examined the role of mobile DNA in bacterial physiology, fitness, or virulence. Kopkowski et al. studied the effect of DNA-binding proteins on the transposition of the IS element upstream of the *bgl* operon in *Escherichia coli*. This operon, which is normally not expressed, is required for the uptake and metabolism of β-glucosides. Insertion of either IS*1* or IS*5* upstream of the *bgl* promoter activates expression of the operon only when the cell is starved in the presence of a β-glucoside, resulting in increased transcription and allowing the cell to survive and support growth using this carbon source. The authors provided evidence that the DNA-binding proteins Crp and IHF exert a positive effect on insertional *bgl* mutations. Their experimental study indicates that through its binding, IHF may exert its effect by altering the DNA conformation of IS*1* and IS*5* at their native locations, rather than by directly influencing transposase gene expression. On the other hand, the cAMP-Crp complex binds upstream of the promoter and presumably alters the local DNA into a conformation that enhances IS insertion. The study of Hussain et al. aimed to remove mobile genetic elements from the genome of *Clostridioides difficile* and to assess the implications of this removal on the biology of the organism. The genome of this pathogen is highly variable and contains mobile DNA elements such as transposons and prophages that influence its biology. Using allele replacement methodology facilitated by CRISPR-Cas9, the authors succeeded in deleting the following DNA elements from two *C. difficile* strains: Tn*5397* (21 kb) and φ027 (56 kb). The growth characteristics of the deleted strains were only altered in minimal medium. The impact of the deletion on conjugal transfer and phage sensitivity was also investigated. The created deletants will be further investigated for the contribution of the targeted mobile DNA elements to the bacterial host's virulence, fitness, and physiology. Related to this study, in the Phage Biology section, Shüler et al. published novel insights into the phage biology of the pathogen *C. difficile* based on the active virome. The authors examined active prophages from different *C. difficile* strains by sequencing and characterizing phage particle-protected DNA following standard cultivation or cultivation under prophage-inducing conditions. Spontaneous prophage release was demonstrated to be common in this pathogen. Fourteen different phages were identified. In addition, the authors showed that enveloped DNA mapped to genomic regions with characteristics of mobile DNA other than prophages, suggesting DNA mobility mechanisms that have not been fully studied in *C. difficile*. Moreover, phage-mediated lateral transduction of bacterial DNA was detected for the first time in this species. Thus, this study contributed to new knowledge regarding prophage activity and phage biology in *C. difficile*.

In the more general field of DNA binding and modification, Helbrecht et al. published in the Microbiotechnology section the characterization of winged helix domain fusion endonucleases as N6-methyladenine-dependent type IV restriction systems. The authors showed that the role of the winged helix domain as a sensor of adenine methylation is widespread in prokaryotes and other potential sensors in modified DNA are also discussed.

In summary, this Research Topic provides a collection of Original Research articles on mobile DNA elements and their contribution to bacterial evolution, such as the acquisition of novel features by their bacterial host to resist environmental or *in vivo* conditions, such as antimicrobial resistance.
